# Traceability and Emission Reduction of Dissolved Inorganic Nitrogen in Minjiang Estuary, China

**DOI:** 10.3390/ijerph181910017

**Published:** 2021-09-23

**Authors:** Chenchen Fan, Peng Zhang, Gangfu Song, Huaru Wang, Bingyi Wang

**Affiliations:** College of Environment and Municipal Engineering, North China University of Water Resources and Electric Power, Zhengzhou 450046, China; fancc2019@163.com (C.F.); zhangpeng2019@ncwu.edu.cn (P.Z.); wanghuaru@ncwu.edu.cn (H.W.); wby13373715778@163.com (B.W.)

**Keywords:** dissolved inorganic nitrogen, macrotidal estuary, sharing coefficient, emission reduction plan

## Abstract

The accumulation of dissolved inorganic nitrogen (DIN) in estuaries has become a global environmental problem. A two-dimensional, hydrodynamic water quality model was constructed in this study to investigate the sources of DIN pollution in the Minjiang Estuary. The concentration response field between the stream input and DIN in the estuary was established by using the surveyed source data of the study area. A sharing coefficient method was used to calculate the contribution percentage of each outfall to derive and propose a reasonable nitrogen reduction plan. The results showed that the input of land-based nitrogen into the Minjiang River contributed more than half of the DIN in the near-shore sea; the middle and upper reaches of the Minjiang River largely influenced the estuary area (38.57%). Conversely, the estuary and the coastline accounted for a smaller proportion of only 5.24%, indicating that an integrated DIN reduction should be implemented in the estuary area of the whole river basin. The model calculations showed that the reduction results, after remediation according to the current national standards for wastewater discharge in rivers, were not satisfactory. Thus, a new scheme is proposed in this paper—the total nitrogen (TN) input from land-based sources into the Minjiang Estuary and from the Shuikou Dam to the Min’an section should be reduced to below 31.64%; simultaneously, the DIN concentration discharged from the Shuikou Dam should be controlled and maintained below 0.5 mg·L^−^^1^ (TN = 0.8 mg·L^−^^1^). These results will provide guidelines for developing strategies for the improvement of DIN and water quality in similar estuaries.

## 1. Introduction

An estuary—a transition zone between river and marine ecosystems—is an extremely fragile and sensitive water body. It is highly affected by land-based pollutants, ebbs and waves, and other phenomena; moreover, its response to the changes in environmental factors differs from that of rivers and oceans [1,2,3,4]. Heightened human activities in recent years have significantly increased the input of dissolved inorganic nitrogen (DIN) in coastal rivers and estuaries, causing a series of problems such as eutrophication, hypoxia, toxic and harmful red tides, and the loss of biodiversity. The affected area and the duration of DIN influence are increasingly expanding [5,6]. A large number of studies have shown that the enormous accumulation of DIN in most estuaries is related to human activities [7,8,9]. Despite government measures for controlling point and nonpoint source pollution, the damage to estuaries and coastal water bodies is worsening at an accelerated rate [10,11]. According to the Bulletin of China Marine Ecological Environment Status [12], sea pollution in the past five years has extended mainly to coastal water bodies such as estuaries and bays, which have accumulated alarming amounts of DIN and labile phosphate. Excessive nutrient enrichment in the coastal and estuary water bodies in China has become a major environmental problem [13,14].

Land–sea integration, i.e., integrated river basin and coastal management, is a key factor in dealing with marine pollution [15,16]. Therefore, subregional calculations of the spatial and temporal flux of pollutants in the upper estuarine basin and their contribution to the water quality of adjacent coasts are important in understanding the coastal estuarine systems and improving the management of nutrient pollution in estuaries [17,18,19]. Many researchers have conducted various studies on nutrient aggregation in estuaries or adjacent coastal areas [20,21,22]. James S. Latimer et al. [23] used a nitrogen loading model to estimate the nitrogen load in the watershed–estuary system by collecting data on the land-use types. They compared the relative magnitude of the nitrogen sources based on the calculated results and proposed measures to meet the nitrogen reduction targets for the estuary. Dan Yu et al. [24] used the Global NEWS-DIN model—a spatially explicit watershed scale model—to quantify the DIN contribution of two tributaries to the estuary by using simple socioeconomic data and model parameters as the inputs; they further derived a future management strategy that could meet the national water quality standards by conducting an abatement scenario analysis. However, estimating the nitrogen load of each type of outfall or stream in order to compare its DIN contribution in an estuary yields relatively inaccurate results. In recent years, the application of sharing coefficient methods based on water quality modelling has gradually increased as an effective way of tracing the pollutants in estuaries [25,26,27,28,29]. Thus, a hydrodynamic water quality model was developed in this study to separately calculate the contribution of individual pollutant sources to estuarine pollution. The nutrient input in the estuary was traced back to individual streams or outfalls. Based on the combination of accurate traceability results and the reasonable reduction potential of pollutants, reducing the nutrient input is considered a reasonable approach to addressing the nutrient build-up from predominant anthropogenic nutrient loadings in estuaries or adjacent coastal areas [30,31].

The Minjiang Estuary has been listed as an estuary with poor water quality many times. In the past five years, the DIN concentration values of the three water quality assessment points in the near-shore waters have not met the water quality standards in most cases. The excessive DIN levels [32] have attracted considerable attention from local authorities, the public, and social media [33]. This study was based on the background that the DIN in the coastal waters of the Minjiang Estuary was more concentrated than the recommended standard value. A two-dimensional hydrodynamic water quality model was constructed based on the results of the 2019 Minjiang River outfall and land-based pollutant surveys, and the sharing coefficient method was applied to calculate the contribution of each outfall and stream in the lower Minjiang River to the DIN concentration in the estuary. We assumed that the nitrogen output from the lower outfall and streams of the Minjiang River would reduce in accordance with the standards of the Nearshore Marine Pollution Prevention and Control Programme. Based on the results of the water quality improvement and weight calculations, a plan to reduce the nitrogen output of the whole Minjiang River basin in order to meet the DIN standard in the Minjiang Estuary is proposed in this paper.

## 2. Research Area

The Minjiang River is the largest river flowing into the sea (total length is 562 km) in the Fujian Province, Southeast China. The Minjiang River basin has a subtropical monsoon climate, with an annual runoff of 1760 m^3^·s^−1^ [34,35]. The Shuikou Dam—as a dividing line between the middle and lower reaches of the Minjiang River—has a controlled minimum ecological flow of 308 m^3^·s^−1^ that sustains the downstream water environment and improves shipping requirements [36]. The lower section of the river divides into the south channel and the north channel at the Wenshanli hydrographic station, converging at the Baiyantan water-level station. Next, it flows in a north-easterly direction through Min’an, where it divides again into two branches, north and south at Tingjiang, bypassing the Langqi island and injecting into the Taiwan Strait from the Changmen Channel and the Meihua Channel, respectively. The Minjiang Estuary, with a regular half-day tidal pattern and an average tidal difference, is 4.1 m in height. The north channel runs through the city of Fuzhou, which receives 85% of the city’s industrial wastewater and nearly 90% of its domestic sewage [37]. The south channel is subjected to low wastewater inputs, with nitrogen sources arriving mainly from the upstream deposits and hard-to-degrade organic matter [36].

**Figure 1:**
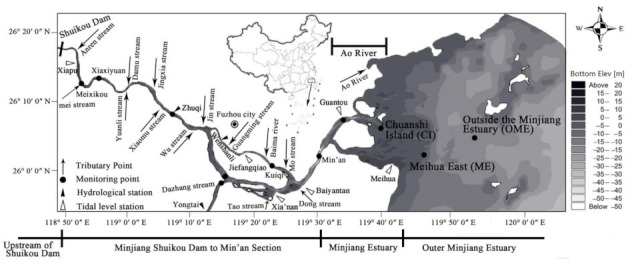
Minjiang River bottom elevation, hydrology, and water quality observation stations along the Minjiang River up to the sea; the regional distribution of DIN and 14 main tributaries are also shown.

Based on factors such as watershed management and pollution control, the DIN-affected area is divided into the Minjiang Estuary (including tributaries, coastal streams, and point source pollution), upstream of Shuikou Dam (upstream of the river), from Minjiang Shuikou Dam to the Min’an section (the lower reach receiving wastewater), the Ao River (the river migrates and spreads to the Minjiang Estuary after entering the sea), and the Outer Minjiang Estuary (Figure 1).

## 3. Pollutant Data Collection and Pre-Treatment

### 3.1. Pollutant Data Collection

**Figure 2:**
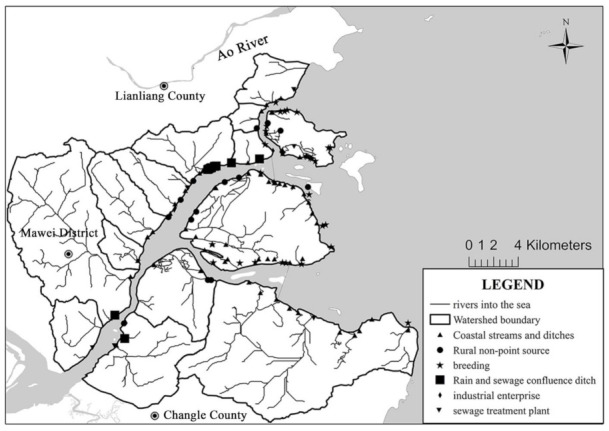
Minjiang Estuary regional catchment area showing the spatial distribution of various types of sewage outfalls after generalization.

The pollution source data of the lower Minjiang River were mainly derived from the survey team’s calculation results on the total nitrogen (TN) loading in 2019. Based on the environmental statistics of Fuzhou, the TN entering the Minjiang River from the Shuikou Dam to Min’an was 21,318 t·a^−1^. Data on land-based pollution sources of the Minjiang Estuary were collected from the second national survey on pollution source conducted by China in 2017. The information on six outfall sources was obtained by the investigation team through a three-level investigation, including industrial enterprises, sewage treatment plants, rain and sewage confluence ditch, breeding, rural nonpoint sources, and coastal streams (Figure 2). Data on the water quality of the Minjiang River and the coastal waters of the Minjiang Estuary were derived from the water quality assessment points (Shuikou Dam, Geyangkou, Xiaxiyuan, Zhuqi, Kuiqi, Dazhangxi, Wanbian, Minan, Guantou, Chuanshi island (CI), Meihua East (ME), and outside the Minjiang estuary (OME) in Figure 1) set up by the Fujian Provincial Department of Ecology and Environment [38], as well as from the results of the team’s seven water quality monitoring surveys on the main stream of the Minjiang River (Geyangkou to Min’an) in 2019. There are three water quality assessment points in the coastal waters of the Minjiang Estuary, namely CI, OME, and ME (Figure 1), where the target concentrations of DIN were 0.3, 0.3, and 0.2 mg·L^−1^, respectively [32].

### 3.2. Determination of Pollutant Conversion Relationship

Given the fact that there is inconsistency in the statistics—the indicators of water quality in the environmental quality standards for surface water and the seawater quality standards are different—the pollutant emission concentration and the river water quality monitoring data should be converted into the water quality concentration corresponding to the ocean. Hence, a linear regression model was used to transform the monitoring data. According to previously reported studies [39,40], the conversion relationships can be changed by several factors such as saltwater intrusion and ebbs and flow of tides. It is unrealistic to obtain detailed water quality data from a large basin. Therefore, the conversion ratio between DIN, NH_3_-N, and TN was obtained by a linear fitting method based on the existing data as:

(1) $$c\left(\mathrm{DIN}\right)\;=\;0.897\;\cdot \;c\left(\mathrm{TN}\right)\;R^{2}\;=\;0.9826$$

(2) $$c\left(\mathrm{DIN}\right)\;=\;1.019\;\cdot \;c\left(NH_{3}-N\right)\;+\;1.820\;R^{2}\;=\;0.9100$$

where $c\left(DIN\right)$, $c\left(NH_{3}-N\right)$, $c\left(TN\right)$ represent concentrations of DIN, NH_3_-N, and TN, respectively (in units of mg·L^−1^)

## 4. Methods

### 4.1. Hydrodynamic Water Quality Model

#### 4.1.1. Basic Equation

The Shuikou Dam–outer seawater environment (covering an area of 160 km) model constructed in this study includes two sub-models: a hydrodynamic model and a water quality model. The investigated area included the coastal open water affected by tides and tidal currents. The two-dimensional unsteady flow continuity and momentum equation were adopted for the following calculations [41,42]:

(3) $$\frac{\partial h}{\partial t}\;+\;\frac{\partial h\bar{u}}{\partial x}\;+\;\frac{\partial h\bar{\nu }}{\partial y}\;=\;hS$$

(4) $$\begin{array}{l} \frac{\partial h\bar{u}}{\partial t}\;+\;\frac{\partial h{\bar{u}}^{2}}{\partial x}\;+\;\frac{\partial h\bar{\nu u}}{\partial y}\;=\;f\bar{\nu }h\;-\;gh\frac{\partial \eta }{\partial x}\;-\;\frac{h}{\rho_{0}}\frac{\partial p_{a}}{\partial x}\;-\;\frac{gh^{2}}{2\rho_{0}}\frac{\partial \rho }{\partial x}\;+\;\\ \frac{\tau_{sx}}{\rho_{0}}\;-\;\frac{\tau_{bx}}{\rho_{0}}\;-\;\frac{1}{\rho_{0}}\left(\frac{\partial s_{xx}}{\partial x}\;+\;\frac{\partial s_{xy}}{\partial y}\right)\;+\;\frac{\partial }{\partial x}\left(hT_{xx}\right)\;+\;\frac{\partial }{\partial y}\left(hT_{xy}\right)\;+\;hu_{s}S \end{array}$$

(5) $$\begin{array}{l} \frac{\partial h\bar{v}}{\partial t}\;+\;\frac{\partial h\bar{uv}}{\partial x}\;+\;\frac{\partial h{\bar{v}}^{2}}{\partial y}\;=\;-f\bar{u}h\;-\;gh\frac{\partial \eta }{\partial y}\;-\;\frac{h}{\rho_{0}}\frac{\partial P_{a}}{\partial y}\;-\;\frac{gh^{2}}{2\rho_{0}}\frac{\partial \rho }{\partial y}\;+\;\\ \frac{\tau_{sy}}{\rho_{0}}\;-\;\frac{\tau_{by}}{\rho_{0}}\;-\;\frac{1}{\rho_{0}}\left(\frac{\partial s_{yx}}{\partial x}\;+\;\frac{\partial s_{yy}}{\partial y}\right)\;+\;\frac{\partial }{\partial x}(hT_{xy})\;+\;\frac{\partial }{\partial y}(hT_{yy})\;+\;h\nu_{s}S \end{array}$$

where t is the time (s), x, y are the cartesian coordinates (m); h is the total water depth (m); η is the surface elevation (m); $\bar{u}$ and $\bar{v}$ are the velocity components in the x and y direction (m^3^·(s·m) ^−1^); f = 2Ω sinφ is the Coriolis parameter (Ω is the angular rate of revolution and φ the geographic latitude); $S_{xx}$, $S_{xy}$, and $S_{yy}$ are components of the radiation stress tensor; $P_{a}$ is the atmospheric pressure (kg/m/s^2^); Q is the water discharge from point sources; $\rho_{0}$ is the reference density (kg/m^3^); g is the gravitational acceleration (m^2^/s); $h\bar{u}\;=\;\int_{-d}^{\eta }udz$, $h\bar{v}\;=\;\int_{-d}^{\eta }udz$, ($u_{s}$,$v_{s}$) is the velocity by which the water is discharged into the ambient water; $\tau_{sx}$, $\tau_{sy}$ are the components of the river bed friction in the x, y direction; $\tau_{bx}$, $\tau_{by}$ are the components of wind field friction in the x, y direction; $T_{xx}$, $T_{xy}$, $T_{yy}$ are the horizontal stress terms.

#### 4.1.2. Basic Equation of Water Quality

The basic equation of convection and diffusion of pollutants in a two-dimensional nonuniform flow is given by [43,44]:

(6) $$\frac{\partial h\bar{C}}{\partial t}\;+\;\frac{\partial h\bar{u}\bar{C}}{\partial x}\;+\;\frac{\partial h\bar{v}\bar{C}}{\partial y}\;=\;h\left[\frac{\partial }{\partial x}\left(E_{x}\frac{\partial }{\partial x}\right)\;+\;\frac{\partial }{\partial y}\left(E_{y}\frac{\partial }{\partial y}\right)\right]\bar{C}\;+\;S$$

where $\bar{C}$ is the concentration of pollutants in the average water depth (mg·L^−1^); $E_{x}$ and $E_{y}$ are diffusion coefficients in the x and y direction (m·s^−2^); S is the source and sink (g·(m^2^·s) ^−1^).

#### 4.1.3. Model Setup

According to the topographic characteristics of the researched area, the model was divided into 53,751 triangular grids. The inner river grid was approximately 120–140 m in size, and the outer sea grid was approximately 350–400 m in size. In addition, the mesh density was increased in some deep and narrow channels. Topographical and river section data were provided by the Survey Center of Fujian Port and Navigation Administration. The water level and discharge were obtained from the hydrological observation stations of the Minjiang River and Hydrology P. R. China (YBHC). The tidal level of the outer sea was calculated and calibrated using TPXO 6.2, which is a global tidal model developed by the Oregon State University. Data on atmospheric boundary conditions such as wind field, temperature, and relative humidity were taken from the China Meteorological Network [45]. The average value of dry and wet sedimentation was adopted in the atmospheric boundary parameters. The values of the sediment release parameters were unevenly distributed in time and space. The upper boundary of the water quality was derived using the daily water quality concentration monitoring values of the nearby Xiongjiang section in 2019. The open boundary of water quality was determined from the values of the conventional water quality monitoring section. All tributaries entered the river in the form of sewage outfalls. For the hydrodynamic boundary, data obtained from the hydrological observation station were used; for the other tributaries without monitoring data in the hydrological station, the catchment area was used to calculate the average runoff. The pollution source data for the Minjiang Estuary region was obtained from the results of detailed pollution investigations. The 51 sewage outfalls not along the Minjiang River were generalized to their corresponding tributaries. The 124 different types of sewage outfalls along the Minjiang River were considered as point sources in the model. The time step was set to 30 s to satisfy the Courant–Friedrich–Levy criterion [46].

#### 4.1.4. Calibration

**Figure 3:**
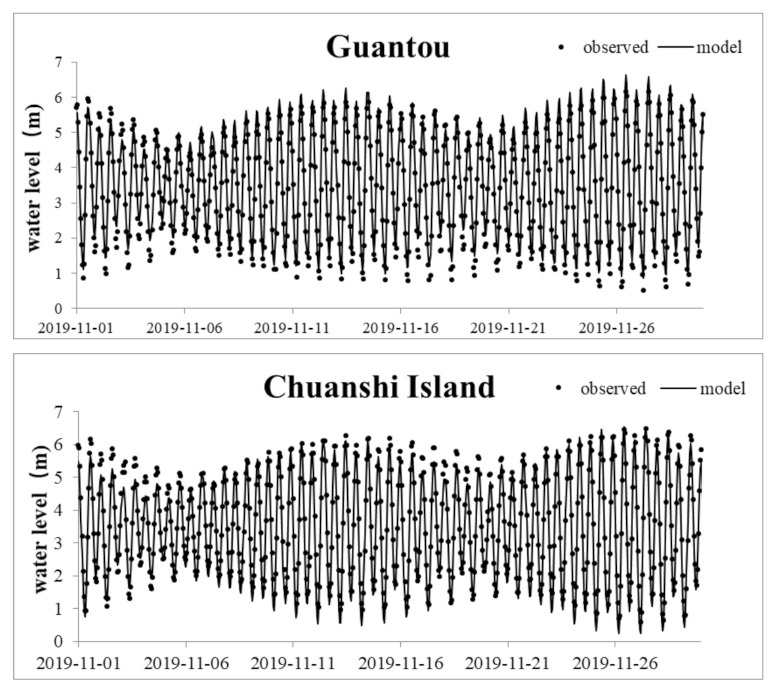
Tide level calibration results for Guantou and CI.

**Figure 4:**
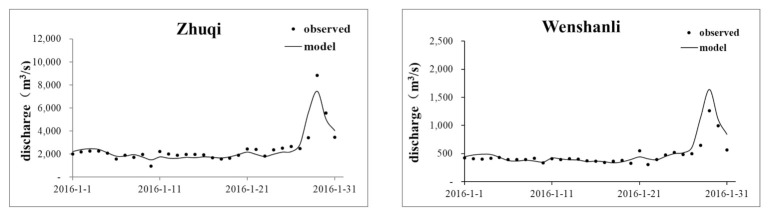
Flow calibration results for Zhuqi and Wenshanli.

**Figure 5:**
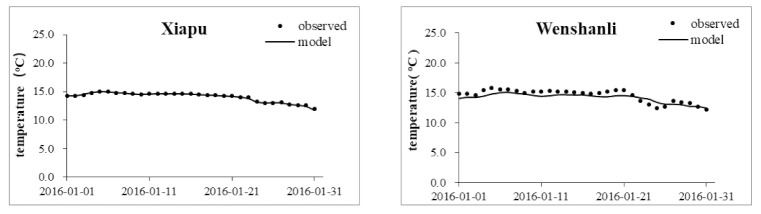
Water temperature calibration results for Xiapu and Wenshanli.

The bottom roughness coefficient, the Smagorinsky coefficient, and the wind field parameters were obtained from the model calibration and verification using the field monitoring values of the model parameters and relevant references. The final roughness parameter of the Minjiang River lower reach was 0.018–0.022 m, and that of the coastal sea was 0.024–0.035 m. The Smagorinsky coefficient was 0.28, and the wind drag coefficient was 0.001–0.0015. Hydrological calibration mainly verified the tide level, flow, and temperature. The data of the hydrological and tidal stations measured in January 2016 were used for the model parameter calibration; the model parameter verification was performed using the measured data from April 2016. Data on the hourly tide levels at the Guantou point and CI station in November 2019 were used for the supplementary verifications. The results of the Minjiang hydrodynamic model tide level verification using the data collected in 2016 are shown in Table 1, and the tide level supplementary verification using the data acquired in 2019 are shown in Figure 3. The flow and temperature calculation results are shown in Figure 4 and Figure 5, respectively. A comparison of the observed and simulated values showed that the average absolute error in the Xia’nan station tide level calculation was 0.20 m, the average relative error in the Zhuqi station flow calculation was 14.16%, and the relative error in the Wenshanli station temperature calculation was 2.93%. The results show that the constructed model can be applied in the development of hydrodynamic simulations of the lower reaches of the Minjiang River and Estuary.

**Figure 6:**
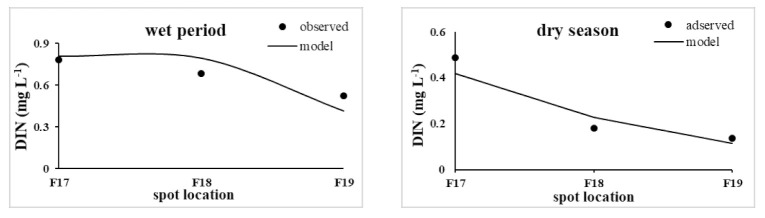
Calibration results of the DIN concentration in the coastal waters of the Minjiang Estuary (F17, F18, and F19 represent the CI, ME, and OME, respectively).

The DIN degradation coefficient, obtained from the model calibration and verification using the field monitoring values of the model-related parameters and relevant references, was 0.07–0.15 d^−1^. The measured data of the three assessment points in the coastal waters of the Minjiang Estuary in 2019 during the dry season were used for the model parameter calibration; the measured data of the wet period in the same year were used for the model parameter verification. The DIN verification results at each assessment point in the Minjiang Estuary are shown in Figure 6 and Table 2. The average relative errors in the DIN concentration at each assessment point were within 21.15%. The errors in water quality in the dry season are relatively large, which is due to the fact that the water quality in the dry season is less affected by land-source pollution flux than that in the wet period, but is more likely to be affected by uncertain factors such as tide currents, atmospheric deposition, and offshore water quality. Among them, the offshore point of the Minjiang Estuary, which is far away, is affected more seriously by uncertain factors. In general, the change in the DIN concentration in the Minjiang Estuary could be successfully simulated using the developed model.

### 4.2. Sharing Coefficient Method

The sharing coefficient method is a type of total control method, which is used to control the total discharge of land-based pollutants [47]. The water quality in an estuary depends on the interaction of various environmental factors such as the location of the sewage outfall, the discharge intensity, and the self-purification capability of the sea [48]. Because the dynamic environmental conditions of the sea area remain unchanged, the response of the seawater quality to the intensity of the pollution source is relatively fixed, yielding a dynamic equilibrium state, which depends only on the location, intensity, discharge volume, and chemical composition of the pollutants [47]. This response relationship is linear and conforms to the principle of linear superposition. Therefore, a functional relationship can be used to describe the relationship between the source intensity and the equilibrium concentration field [49,50]:

(7) $$C_{i}\;=\;P_{i}S_{i}$$

where $S_{i}$ is the emission intensity of the $i^{th}$ point source, $C_{i}$ is the equilibrium concentration value obtained under the sole action of the $i^{th}$ point source, and $P_{i}$ is the response coefficient related to the $i^{th}$ point source.

The equilibrium concentration field formed under the joint action of multiple pollution sources is equivalent to the linear superposition of the concentration fields of individual pollution sources:

(8) $$C\;=\;\sum_{i\;=\;1}^{n}C_{i}$$

where n is the number of point sources.

(9) $$R_{i}\;=\;C_{i}/C$$

where $R_{i}$ (percentage) represents the influence on the sea area of the first pollution source in the overall pollution influence, which quantitatively reflects the contribution of each point source in the dynamic environmental interaction system.

Assuming that $C_{s}$ and $C_{si}$, which represent the concentrations of a pollutant and the i*^th^* point source, respectively, meet the water quality control objective, and that the contribution of the concentration of each point source to the control point is constant, we may write the following equations:

(10) $$C_{si}\;=\;F_{i}C_{s}$$

(11) $$Q_{si}\;=\;C_{si}/P_{i}$$

where $Q_{si}$ is the allowable emission from the i*^th^* point, calculated by using the sharing coefficient method at the control point; thus, the total allowable emission for the area is the sum of the minimum allowable emissions for each point source, calculated from the control point.

(12) $$Q\;=\;\sum_{i\;=\;1}^{n}minQ_{si}$$

In the cases where the water quality standards are exceeded, the discharge from certain sources should be reduced. If the actual volume of discharge is S, then the equation can be written as follows:

(13) D = S−Q

where D is the total reduction. Clearly, when  D>0, the actual discharge exceeds the permitted value and should be reduced. When D < 0, it means that the actual discharge from the outfall is less than the permitted discharge and need not be reduced, although the value of D in this case indicates that the outfall is still influenced by the residual environmental effects.

## 5. Results and Discussion

### 5.1. Calculation of Sharing Coefficient

**Figure 7:**
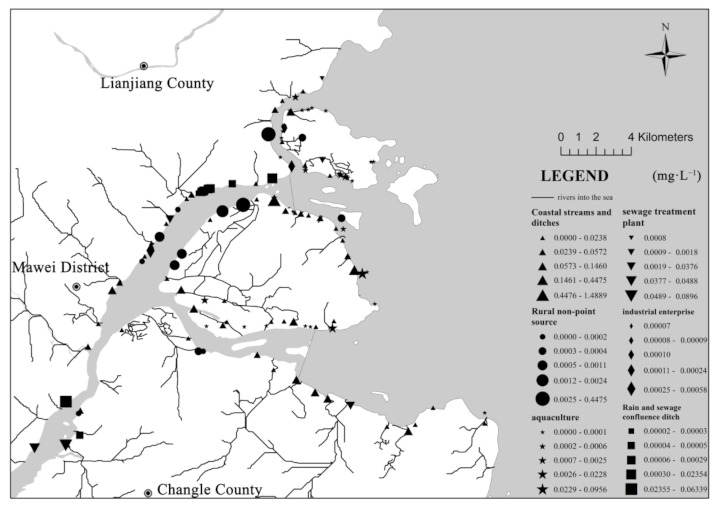
Proportion of DIN contribution from various types of sewage outlets in the land area of the Minjiang Estuary.

**Figure 8:**
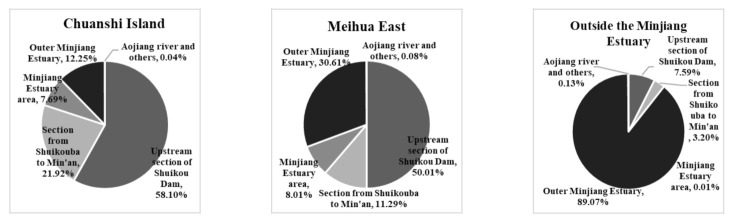
Contribution of each affected area to the DIN concentration in the water quality observation point in the coastal waters of the Minjiang Estuary.

In this study, a high-water period with high fluxes of pollutants from land-based sources was selected to calculate the sharing coefficient. The nitrogen concentration field, formed by each outfall and stream, was individually calculated for the assessment points in the Minjiang Estuary using the collated and investigated source information. The calculated contribution weight of each outfall in the Minjiang Estuary region is shown in Figure 7. The contribution weights of each area to the three assessment points were obtained by summing the weights of the sewage outlets and streams in each affected area. (Figure 8).

The distribution of the DIN contribution weights showed that the nitrogen output of the outfalls and tributaries along the Changmen Channel was higher than that of the Meihua channel. The CI water quality assessment point of the Minjiang Estuary is located near the entrance of the Changmen Channel, which is actually affected by the tidal reciprocating flow. The DIN concentration in the water was found to be high owing to the accumulation of pollutants and poor water exchange capacity. The influence of land-based nitrogen output in the Minjiang Estuary on the DIN in the near-shore area was relatively small (5.24%); the streams and ditches along the river (sea) account for more than half (55.05%) of this contribution. There were 54 streams along the Minjiang Estuary, of which six had a flow of more than 1000 m^3^·d^−1^. The area around the Minjiang Estuary had a large economic system and a dense population. However, the urban sewage collection rate was 62%, while the takeover rate in the non-built-up areas was only 1.8%. The discharge of rural domestic wastewater and agricultural wastewater into the basin introduced a large amount of nitrogen into these streams. In addition, there were many industrial sewage outlets along the stream. The proportion of the rainwater to the sewage confluence ditch was 2.98%, which was due to the high concentration of nitrogen sources in the river; however, its flow was related to the rainfall. Thus, it is proposed that the initial rainwater should flow into the sewage network as a potential treatment measure. The proportion of sewage outlets of the industrial enterprises along the river was the smallest (2.51%) because the direct industrial discharge into the river (sea) has been strictly controlled by the state in recent years; simultaneously, a large number of substandard outlets have been banned and renovated; there were only two industrial enterprises with large NH_3_-N loads in the region.

In general, the rate of DIN contribution in the Minjiang Estuary affected by land-based pollutants showed a decreasing gradient distribution from the estuary to the open sea. CI is located at the estuary of the Changmen Channel, i.e., the main channel of the Minjiang River, with a sharing rate of 87.85% influenced by a land-based source. The ME point is in a protected area of the wetland system; thus, the assessment result should comply with the Class II seawater quality standard (DIN = 0.2 mg·L^−1^). Although the discharge of the Meihua channel is less than that of the Changmen Channel, the DIN contribution rate in the Minjiang Estuary is 30.61%, which is nearly four times more than that of the CI (7.69%). OME—the farthest assessment point from shore—benefits from the high-quality water of the outer sea; the water quality in the OME was the best among the three points, and the DIN concentrations failed to meet the standards only in the dry season during the whole year. The DIN contribution of the upstream area of the Minjiang Dam was the largest among the affected areas (38.57%) in the three assessment points. Due to the high flow of the upper Minjiang River, a large number of nitrogen sources were washed and diffused into the Minjiang Estuary. Like other estuaries, the Minjiang Estuary was affected by the tidal reciprocating flow and thus had poor water exchange, resulting in pollution retention in the estuary, which is the main reason for DIN accumulation in the offshore waters.

### 5.2. DIN Emission Reduction Plan

The reduction of the nitrogen output of sewage outfalls and streams in the estuary is controlled according to the management requirements of the sewage outfall and river water quality. If the three assessment points cannot not meet the corresponding requirements, then more stringent emission reduction measures should be considered.

#### 5.2.1. Pollution Source Reduction Plan in the Estuary

**Figure 9:**
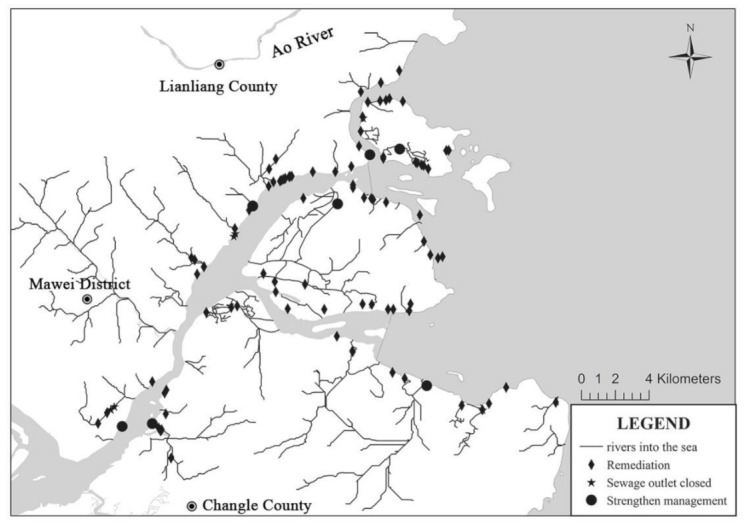
Distribution of sewage outfalls for the rectification in the Minjiang Estuary.

**Figure 10:**
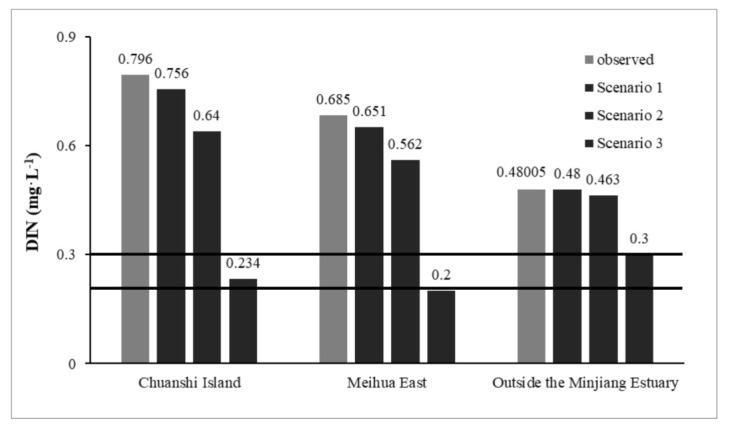
Prediction results after reduction of pollution sources in the land area of the Minjiang Estuary.

In accordance with the requirements for the regulation of sewage outfalls directly or indirectly connected to the sea in the Pollution Prevention Plan for Coastal Waters [51], the 175 surveyed sewage outfalls were classified into three categories: banned according to the law, strengthened management, and improvement (Figure 9). In the regulation of outfalls, four outfalls that severely exceeded the standards or could not be rectified were banned in accordance with the law, while the management was strengthened in two industrial enterprises and six sewage plants in accordance with the provisions of the Marine Environmental Protection Law of the People’s Republic of China. Twenty-five outfalls that exceeded the standards for aquaculture discharge were strictly controlled to meet the corresponding industry standard for DIN concentration, i.e., below 1 mg·L^−1^. Four outfalls that exceeded the standards for freshwater pond culture were controlled to reach the industry standard for TN discharge concentration of less than 5 mg·L^−1^. Rainwater and sewage diversion remedies should be implemented in the built-up areas of the city; twenty-one outfalls in the non-built-up areas could not be connected to the sewage network in order to bring them up to the comprehensive sewage standard of 25 mg·L^−1^ TN or less. Desilting and other remediation works were carried out in 38 coastal streams and ditches, whose monitoring results exceeded the standard, in order to improve the water quality and bring it to the level of the TN standard (1 mg·L^−1^). In this study, the water quality was calculated using the established hydrodynamic water quality model after pollution control in the Minjiang Estuary, while keeping the other hydrological water quality conditions constant during the wet period (Figure 10).

From the predicted results, it can be seen that the DIN concentration was reduced from 0.796 to 0.756 mg·L^−1^ at CI and from 0.685 to 0.651 mg·L^−1^ at ME, with a water quality improvement rate of only 5% (approximately). However, the DIN concentration at the OME showed almost no change. After the nitrogen sources in the Minjiang Estuary area were reduced according to the set emission reduction scenarios, the overall DIN in the nearshore waters were reduced as compared to the status quo values [32]. However, the DIN still severely exceeded the standard at CI, ME, and OME.

#### 5.2.2. Emission Reduction Plan of Pollution Sources in Each Division

In scenario 1, all the pollution sources in each area were controlled to meet the current national control standard for nitrogen sources according to the requirements of the environmental function planning of offshore waters in Fujian Province and the environmental function zoning of the surface water in Fuzhou City. The water quality of the lower discharges of the Shuikou Dam and Ao River was controlled to 1.0 mg·L^−1^ for TN, and those of the 14 tributaries in the area spanning the Shuikou Dam and Min’an were controlled to 1.0 mg·L^−1^ for TN. Each riverine outfall was set to meet its corresponding industry effluent discharge standards. The Minjiang Estuary near-shore sea water quality was calculated after each division of the region to meet the current control standards for reduced emissions. The corresponding results are shown in Figure 10.

The calculation results show that the DIN concentrations at the CI, ME, and OME points were reduced from 0.796, 0.685, and 0.480 mg·L^−1^ to 0.640, 0.562, and 0.463 mg·L^−1^, respectively, with water quality improvement rates of 19.59%, 17.95%, and 3.54%, respectively. The DIN concentrations at all the three points were significantly reduced as compared to those in scenario 1; however, the water quality standards [32] of the corresponding assessment points were still not met. This was mainly caused by the inconsistent water quality standards in rivers as well as lakes and seawater. Thus, it is necessary to strictly control the water quality in the upper reaches of the Minjiang River to reduce the influence of the area above the Shuikou Dam on the DIN in the near-shore waters of the Minjiang Estuary.

#### 5.2.3. Emission Reduction Plan to Meet the DIN Concentration Standard

In scenario 2, the nitrogen load in each affected area of the Minjiang was reduced in equal proportions by taking the treatment of urban domestic pollutants into account, according to the calculation results of the DIN pollution contribution in each divided area. The final reduced TN (DIN) for each region to meet the water quality requirements of the three points in the Minjiang Estuary was obtained via trial calculations, which were performed using the proposed model. The calculations showed that the concentration of DIN in the lower waters of the Shuikou Dam need to be controlled to 0.5 mg·L^−1^ (TN = 0.8 mg·L^−1^) or less, and the discharge of TN pollutants in the area from Shuikou to Min’an need to be reduced by 31.64%, i.e., from the original value of 19,912 t·a^−1^ to 13,611 t·a^−1^. Furthermore, the concentration of DIN in the Ao River also needs to be controlled to 0.5 mg·L^−1^ (TN = 0.8 mg·L^−1^) or less, and that each subbasin that exceeds the surface water quality standard needs to be controlled to less than 0.61 mg·L^−1^ (TN = 0.82 mg·L^−1^). Moreover, the total amount of TN pollutants in the land outfall of the Minjiang Estuary should be reduced by 31.64%. The established water environment model calculations suggest that the concentration of DIN in the near-shore waters of the Minjiang Estuary can be improved to meet the assessment standard. Therefore, it is recommended that the existing water quality requirements in the upper reaches of the Minjiang River should be appropriately raised; simultaneously, a comprehensive water environment management project for the control and reduction of nitrogen pollution in the entire Minjiang River basin should be proposed and implemented in the 14th Five-Year plan of the Fujian Province.

## 6. Conclusions

A two-dimensional hydrodynamic water quality diffusion model was constructed in this study to investigate the main factors that continuously lead to an excessive increase in DIN concentration, beyond the recommended standard, in the coastal waters of the Minjiang Estuary. A comparison between the hydrological and water quality monitoring values showed that the model calculations could be used to derive the requirements necessary for ameliorating the hydrodynamic water quality of the Minjiang river. The pollution concentration field formed under the action of a single pollution source could be calculated accurately using the water quality model based on the sharing coefficient method, tracing the source of DIN in the Minjiang Estuary. The results showed that land-based pollution was the main reason for the continuous increase in DIN concentrations in the affected areas of the Minjiang Estuary. Among them, the upper reaches of the Shuikou Dam accounted for the largest proportion, i.e., 38.57%, which was directly related to the pollutant flux in the upper reaches of the Minjiang River. Pollution in the land area of the Minjiang Estuary accounted for a relatively small percentage of 5.24%.

The DIN concentration after emission reduction was simulated based on the three assessment points, reflecting the change of DIN in the coastal waters of the Minjiang Estuary in accordance with the current pollutant control standards in China. The land pollution prevention remedies according to the Coastal Pollution Prevention and Control Plan in the Minjiang Estuary are not sufficient. The local government can propose appropriate measures to reduce the TN pollutants by 31.64% and control the DIN concentration upstream of the dam to less than 0.5 mg·L^−1^ (TN = 0.8 mg·L^−1^), based on the results of our study for attaining the standard value in each divided area. These measures, if implemented, can significantly improve water quality and reduce eutrophication in the estuary.

## Figures and Tables

**Table 1:** Statistics of the tide level and flow simulation errors obtained for the 2016 data.

Tide Level Error	Zhuqi	Wenshanli	Xianan	Baiyantan	Guantou
mean absolute error (m)	/	0.25	0.20	0.22	0.22
mean relative deviation (%)	14.16%	13.06%	/	/	/

**Table 2:** Calibration error in the DIN concentration at the monitoring points in the coastal waters of the Minjiang Estuary.

	Hydrologic Stations	Degradation Coefficient of the DIN		
Period		CI	ME	OME
wet period		3.70%	16.51%	20.38%
dry season		16.91%	20.70%	21.15%
